# Case report: A novel *STXBP1* splice variant and the landscape of splicing-involved *STXBP1*-related disorders

**DOI:** 10.3389/fneur.2023.1146875

**Published:** 2023-03-28

**Authors:** Haiping Wang, Xiuli Chen, Zhanli Liu, Chen Chen, Xin Liu, Mingwei Huang, Zhuying Zhou

**Affiliations:** ^1^Department of Neurology, Hangzhou Children's Hospital, Hangzhou, China; ^2^Aegicare (Shenzhen) Technology Co. Ltd., Shenzhen, China

**Keywords:** *STXBP1*-related disorder, Ohtahara syndrome, epilepsy, splice variant, functional study, RT-PCR, intron retention, genotype-phenotype relationship

## Abstract

*STXBP1* variants are one of the most common genetic causes of neurodevelopmental disorders and epilepsy, wherein *STXBP1*-related disorders are characterized by neurodevelopmental abnormalities in 95% and seizures in 89% of affected patients. However, the spectrums of both genotype and phenotype are quite wide and diverse, with a high baseline variability even for recurrent *STXBP1* variants. Until now, no clear genotype–phenotype correlations have been established and multiple disease mechanisms have been proposed for *STXBP1*-related disorders. Without an ascertained disease cause for many cases of *STXBP1* variants, it is challenging to manage this disease in an effective manner and current symptom-based treatments are focused on seizure control only, which has a minimal impact on global development. A novel *STXBP1* canonical splice variant, NM_001032221.4:c.578+2T>C, was reported in this study, together with detailed documentation of disease manifestations and treatment management. Further RNA expression analysis revealed abnormal intron retention and possible production of truncated STXBP1 proteins as a likely pathogenic mechanism. More importantly, the landscape of previously understudied *STXBP1* splice variants and functional investigations was assessed for the first time to provide a context for the discussion of the complicated genotype–phenotype relationship of *STXBP1*-related disorders. Future cases of this disorder and a deeper mechanism-based understanding of its pathogenic cause are required for precision medicine and better disease management.

## Introduction

Disease-relevant *STXBP1* variants are one of the most common genetic causes of neurodevelopmental disorders and epilepsy, wherein *STXBP1*-related disorders are characterized by neurodevelopmental abnormalities in 95% and seizures in 89% of affected patients (1, 2). However, the overall phenotypic spectrum of *STXBP1*-related disorders is quite broad. According to one recent comprehensive profiling, the patients could be grouped into several categories, which were early onset epileptic encephalopathy (EOEE), Ohtahara syndrome (OS), West syndrome (WS), other developmental and epileptic encephalopathies (other DEE), neurodevelopmental disorders (NDD), and atypical Rett syndrome (2). The group of EOEE included patients with a seizure onset within 3 months of age and clinical manifestations of developmental and epileptic encephalopathy. However, a more specific category of EOEE (such as OS or WS) would be assigned if possible. The OS group had tonic seizures and suppression-burst electroencephalogram (EEG) in addition to EOEE, whereas the WS group presented with infantile spasms as the first seizure presentation. The group of other DEEs showed DEE which was not categorized as EOEE, OS, or WS. However, if patients showed developmental abnormalities, seizures (if any) could be controlled with medicine and there were no signs of epileptic encephalopathy (significant EEG findings), they would be classified as NDD. In addition, the group with atypical Rett syndrome had developmental abnormalities and Rett-like features.

*STXBP1* encodes the syntaxin-binding protein 1 (STXBP1, also known as Sec1/Munc18-1), which is well characterized in its interaction with syntaxin-1 and regulation of synaptic vesicle and neurotransmitter release (1, 3). In addition, STXBP1 also binds other protein partners and involves in non-synaptic processes (such as Golgi transport and intracellular trafficking), suggesting a broad involvement in cellular activities and thus providing a possible explanation for the diverse phenotypes of *STXBP1*-related disorders (4). Disease-related *STXBP1* variants include missense, nonsense, splice-site, frameshift, deletion, and other variants, spanning the full spectrum of genetic mutations (1, 2, 4). Multiple pathogenic mechanisms have been proposed for *STXBP1*-related disorders, such as haploinsufficiency, dominant negative effects, and gain-of-function molecular consequences (3, 5, 6). However, due to a high baseline variability, no significant phenotypic similarity or discrete phenotypic subgroups emerged for recurrent *STXBP1* variants and mutation hotspots (2). In addition, no clear genotype–phenotype correlations have been identified so far from several large-scale analyses (2, 4, 7).

In this study, we identified a novel heterozygous *STXBP1* splice variant from a patient with OS and assessed the splicing defect with *ex vivo* RNA expression analysis of patient blood samples. We discussed the genotype–phenotype relationship within the context of previously reported *STXBP1* splice variants and functional investigations. Future cases of this disorder and a deeper mechanism-based understanding of its genotype–phenotype relationship are required for precision medicine and better disease management.

## Case presentation

**Figure 1:**
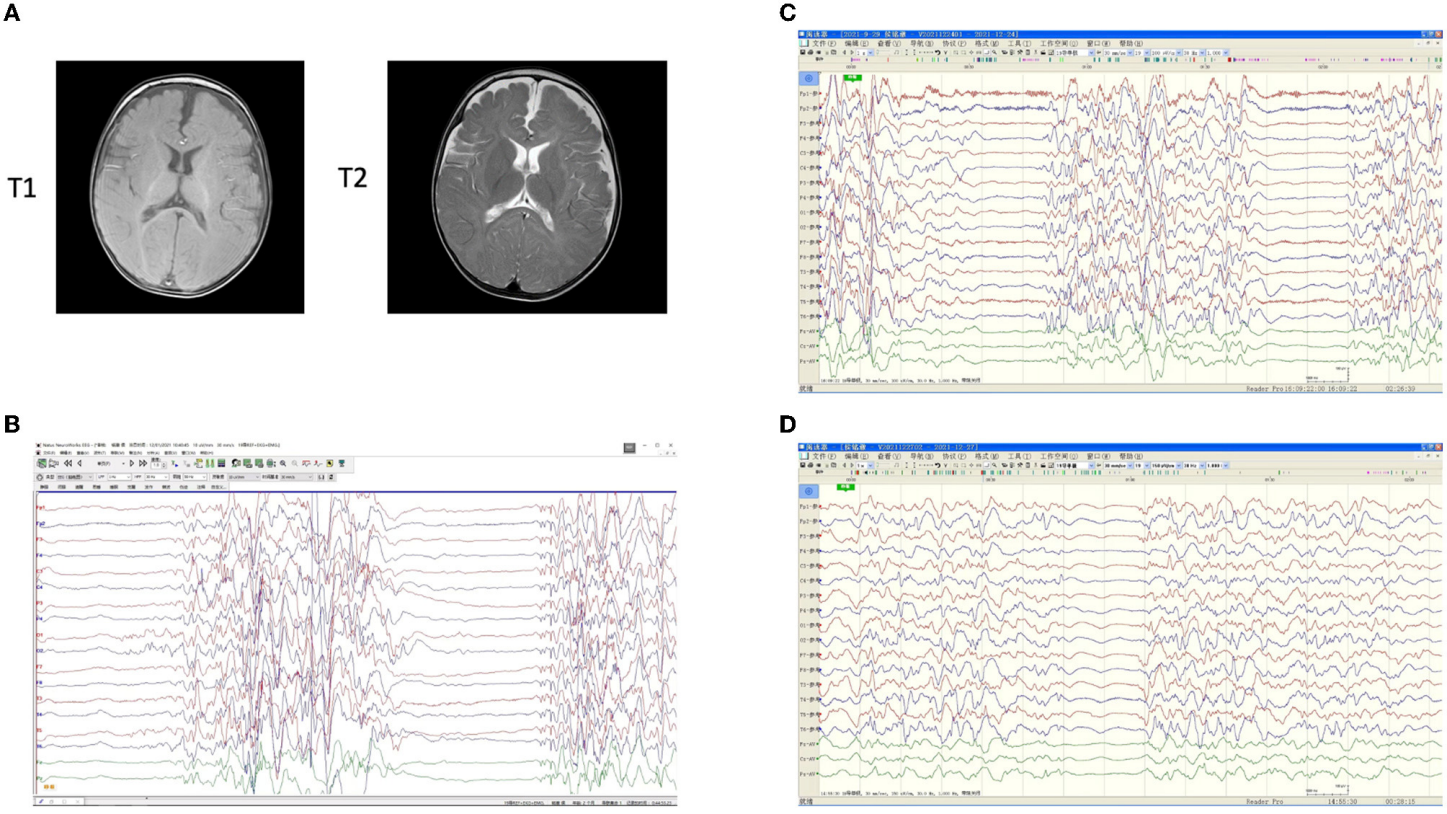
Imaging examinations of the patient. Brain magnetic resonance imaging (MRI) showed no obvious abnormality at the age of 6.5 months **(A)**. T1-weighted and T2-weighted images were consistent. Electroencephalogram (EEG) results before and after antiepileptic medications **(B**–**D)**. **(B)** A burst-inhibition pattern was observed before any medication at 2 months. **(C)** With a treatment of oral adrenocorticotropic hormone (ACTH), topiramate, and valproic acid, the intensity of the burst period was significantly weakened, but the burst-inhibition pattern was still obvious, and their durations remained relatively long. In addition, a small amount of bilateral discharge and an extremely asymmetric background were observed. **(D)** After treatment of prednisone, topiramate, levetiracetam, and midazolam, there were very few burst periods and reduced inhibition periods, with significantly shorter durations at 3 months. A small amount of 4–6c/sθ activity was visible and distributed. Distinguishable focal discharges in the background, apparent asymmetry, and a tendency for a high degree of arrhythmia were also observed.

One 15-month-old male patient presented with neonatal-onset, repeated seizures, and progressing developmental delay (Table 1 and Supplementary Table 1). At 2 months of age, the patient was admitted to the hospital and a clinical diagnosis of OS was made based on the burst-suppression EEG result (Figure 1B). With the administration of adrenocorticotropic hormone (ACTH), topiramate, and valproic acid, the intensity of the burst period was weakened, but the burst-inhibition pattern was still obvious, and their durations remained relatively long by EEG (Figure 1C). At 3 months of age, with a treatment of prednisone, topiramate, levetiracetam, and midazolam, EEG showed very few burst periods, reduced inhibition periods, and significantly shorter durations (Figure 1D). During follow-ups at 4 months (18 weeks) and 6 months (26 weeks), the epileptic seizures were under control, and EEG findings significantly improved, but the development remained markedly behind (Table 1 and Supplementary Table 1). Brain magnetic resonance imaging (MRI) showed no obvious abnormality at the age of 6.5 months (Figure 1A).

**Table 1:** Disease timeline and therapeutic interventions/outcomes.

**Age**	**Clinical descriptions**
**At birth**	The patient was born at 40 weeks by natural delivery, with a weight of 3320 g and height of 50 cm, passing newborn screenings of foot blood and hearing. The mother of the patient had hypothyroidism and was treated with oral Eucalyptus during pregnancy. The patient was breastfed for 1 month after birth, and then on artificial feeding. Immunization was performed according to local regulations, without any noted adverse reactions. His parents were healthy and there was no family history of epilepsy or other genetic disorders.
**Shortly after birth**	Clustered seizures like nodding and hugging multiple times a day, more than 10 at a time and each lasting 1–2 min.
**1.5 months**	Bilateral or unilateral limb tonic seizures or tonic-clonic seizures occurred, more than 10 times a day and each time lasting 2–10 s.
**2 months**	**Hospital admission** and diagnosis of Ohtahara syndrome based on burst-suppression electroencephalogram (EEG). Physical examination at admission revealed consciousness, poor response, ability to suck, inability to eye-track or to hold the head up. Eye examinations revealed slight esotropia, pupils on both sides of equal size and circle, diameters at about 2 mm, and with light reflex. Further tests showed stable breathing, inspiratory depression in the suprasternal fossa, normal heart and lung auscultation, soft abdomen, and no palpable enlargement of the liver or spleen under the ribs. Besides, the neck was soft, the muscle strength and muscle tone of the limbs were low, the bilateral Babinski sign was negative, and there was a livedo about 2 cm*2 cm on the back. Blood biochemical examinations and genetic metabolism screening also revealed nothing remarkable in lactate, blood ammonia, ceruloplasmin, thyroid function, blood liver and kidney function, electrolytes, and blood sugar. Treatment with oral Adrenocorticotropic Hormone (ACTH), topiramate, and valproic acid.
**2 months and 3 weeks**	Poor outcome (EEG) and severe pulmonary infection resulted in admission to the Pediatric Intensive Care Unit; ACTH ended and prednisone acetate tablets added; topiramate and valproic acid continued.
	One *STXBP1* variant identified through genetic testing; oral levetiracetam and midazolam micropump added, prednisone acetate and topiramate continued, valproic acid reduced and withdrawn.
**3 months**	Seizures under control (EEG) and reduced to 1–3 times a day; midazolam withdrawn and oral clonazepam tablets added (both midazolam and clonazepam are benzodiazepines).
	Discharge from hospital with prednisone acetate tablets, topiramate, levetiracetam oral liquid, and clonazepam tablets; seizures under control.
**4 months**	Seizure attacks were in the form of binocular staring, with or without rigidity of both upper limbs, 3–4 times a day.
**5 months**	The symptoms were similar to those at 4 months, with reduced seizure attacks at once every 2–3 days and improved EEG; prednisone acetate tablets withdrawn and topiramate gradually reduced, whereas oral levetiracetam liquid and clonazepam continued.
**6 months**	Seizures were under control and occurred once every 10 days. EEG findings significantly improved, but the development was still markedly behind. The muscle strength and muscle tone of the limbs were low.

## Genetic diagnosis

**Figure 2:**
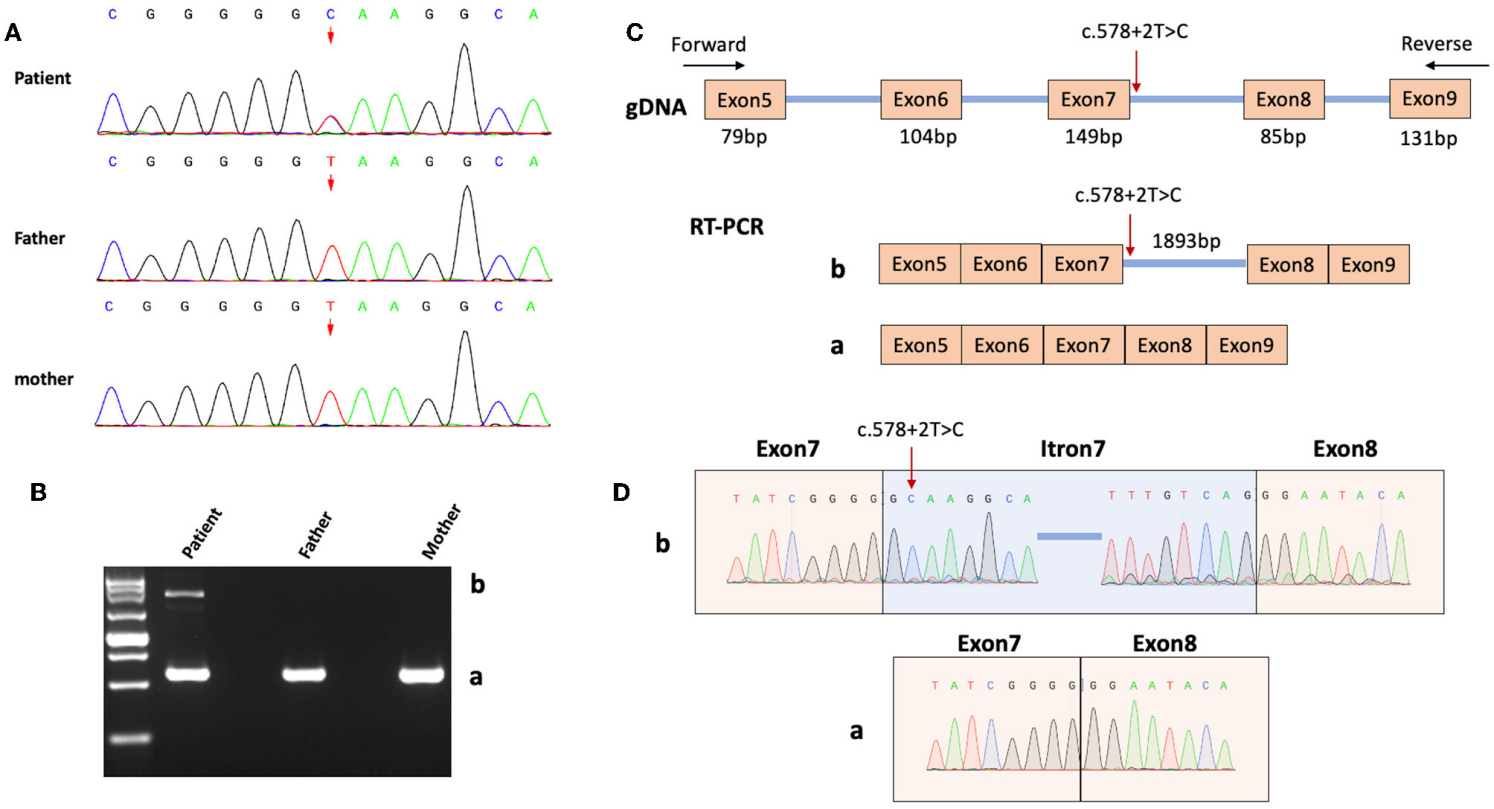
Genetic diagnosis and RNA analysis. **(A)** Sanger sequencing results of the patient and his parents. The arrow indicates the genomic location (according to the human genome assembly GRCh37 / hg19) of the heterozygous variant NM_001032221.4:c.578+2T>C. **(B)** RT-PCR product analysis by agarose gel electrophoresis. A normal band (Band **a**) was observed in both the male patient and controls (his parents), whereas an extra band (Band **b**) was observed in the patient only. **(C)** A diagram showed the design of PCR primers for cDNA amplification and the contents of the two bands in B (Bands **a** and **b**). Red arrow indicates the variant site. **(D)** Sanger sequencing revealed the sequences of Bands **a** and **b**.

A pathogenic heterozygous variant, NM_001032221.4:c.578+2T>C, was identified in the *STXBP1* gene from the patient through whole genome sequencing. This variant is located at a canonical splice donor site and is predicted to result in abnormal splicing of *STXBP1* mRNA. To date, this novel variant has no minimum allele frequency documented in the Reference Population Gene Frequency Database (gnomAD) and has not been reported in the Clinvar database. By Sanger sequencing analysis, this mutation was not identified in the patient's father or mother (Figure 2A). According to the American College of Medical Genetics and Genomics (ACMG) guidelines, this novel *de novo* variant is classified as pathogenic. Together with the clinical findings described earlier, a final diagnosis of STXBP1 encephalopathy with OS was made.

## RNA analysis for splicing defect of our *STXBP1* variant

*STXBP1* c.578+2T>C is one canonical splice variant, which usually leads to exon skipping, intron retention, and/or the activation of an alternative cryptic splice site (8). RNA expression analysis was carried out on patient blood samples using a method published earlier (9). Briefly, RNA was first extracted from blood samples collected from the patient and his parents as controls. Complementary DNA was then obtained and one pair of primers was designed to amplify regions of interest, which were separated by agarose gel electrophoresis. Bands of interest were gel extracted and sequenced.

As shown in Figures 2B, C, the normally spliced mRNAs from both parents generated a PCR product of 570bp (Band **a**) that contains 4-bp at the 3' end of Exon4, 79-bp Exon5, 104-bp Exon6, 149-bp Exon7, 85-bp Exon8, 131-bp Exon9, and 18-bp at the 5' end of Exon10. However, samples from the patient with heterozygous *STXBP1* c.578+2T>C showed two PCR products, of which the normal one was Band **a** and an abnormal one (2463bp, Band **b**) contains the additional 1893-bp Intron7. Moreover, sequencing results of Band **b** showed that the second nucleotide of the retained Intron7 is the variant T, rather than the normal C, indicating that the abnormally spliced transcript was from the variant allele as a result of the splicing defect (Figures 2C, D). The translation of the abnormal transcript would lead to a premature termination codon within the retained Intron7 and therefore a truncated STXBP1 protein.

## The overall profile of *STXBP1* canonical splice variants

Our identified variant, *STXBP1* NM_001032221.4:c.578+2T>C, is located at a canonical splice site and leads to abnormal splicing. In general, canonical splice variants, within 2bp of exon–intron junction, are widely annotated as “loss of function” (LoF) variants and are known to be strong diagnostic candidates in LoF disorders (10). For example, +2T>C variants have been frequently reported to cause human genetic disease and are routinely scored as pathogenic splicing mutations. However, it was recently demonstrated that diverse molecular outcomes exist and such +2T>C variants in human disease genes may not invariably be pathogenic (11, 12).

To obtain a comprehensive understanding of the genotype–phenotype relationship for *STXBP1* splice variants, we compiled a list of 54 canonical and 203 non-canonical splice variants (Supplementary Table 2) from the Clinvar database. We evaluated those variants through spliceAI, a deep neural network that accurately predicts splice sites based on pre-mRNA sequence, which proves to be a highly accurate and informative prediction tool for potential splicing changes (10, 12, 13). Most canonical splice variants (44 out of 54) cause frameshift insertion or deletion and thus disruptive changes in *STXBP1* expression. Out of the 44 frameshift splice variants, 12 are associated with early infantile epileptic encephalopathy with suppression bursts, 16 are associated with developmental and epileptic encephalopathy, whereas the rest do not have specified clinical conditions.

Because many of the variants reported by Clinvar do not have a phenotypic description available for in-depth assessment (only general disease category provided), clinical information from the comprehensive list of 534 individuals with *STXBP1*-related disorders published in 2022 (2) was referenced for phenotype analysis. A brief survey of multiple canonical splice variants spanning the full length of *STXBP1* pre-mRNA (Supplementary Table 3) showed that the majority of them correlated with severe phenotypes and early disease onset in the patients (12 at more than 2 months and only one at 14 months), suggesting that *STXBP1* is highly sensitive to decreased amount of expression, consistent with its high probability of loss-of-function intolerance and predicted probability of haploinsufficiency (14).

For the ten canonical splice variants of *STXBP1* that were predicted to result in in-frame splicing changes, it is reasonable to speculate that their consequent less disruptive changes would lead to less severe phenotypes in general than those with frameshift changes. Previously published data (2) were utilized for this analysis (Table 2). It seems that smaller deletion (one case of c.795-2A>T, 12bp deletion, and moderate delay with seizure onset at 2.5 months) causes phenotypes less severe than larger deletion (two cases of c.795-2A>G, 108bp deletion, severe delay with seizure onset at 0.5 or 0.35 month). Different changes at the same splice site (c.795-2A>T/G) could cause different molecular consequences enough for significant clinical differences, supporting a previous notion that the functional effect of splicing variants is on a continuum rather than binary (10). Similarly, c.1030-1G > A is predicted to result in skipping of the whole 81-bp Exon13, compared to a deletion of only 27bp caused by c.1030-1G>T. More drastic differences likely exist between c.1462-2A>G (12bp in-frame deletion) and c.1462-2A>T (86bp frameshift deletion), but unfortunately, no clinical data are available to assess the genotype–phenotype relationship. More well-documented cases and experimental characterizations of the exact molecular defect are needed to draw a more convincing conclusion since only a small number of splice variants were currently available with experimental characterization and SpliceAI predictions were used as a proxy for functional evaluation.

**Table 2:** Canonical splice variants of *STXBP1*.

**Variant**	**Patient ID**	**Disease categories**	**Disease onset/month**	**SpliceAI prediction**
c.578+1G>A	STX_32139178_Patient_Infantile22	EOEE	3	Frame-shift
c.578+1dupG	STX_CHCO_01	EOEE	0.1	Frame-shift
c.578+2T>C	This study	OS (EOEE)	**0**	Frame-shift
c.169+2T>C	STX_31344879_Patient3	Atypical Rett Syndrome	2	Frame-shift
c.1249+2T>C	STX_HSJD_Patient_9	EOEE	0.13	Frame-shift
	STX_25631041_case_report	OS	0.49	
c.1359+1G>A	STX_P_28	EOEE	72	Frame-shift
	STX_HSJD_Patient_7	EOEE	1	
	STX_26865513_Patient_13	EOEE	4	
c.1702+1G>A	STX_31344879_Patient5	Atypical Rett Syndrome	0.5	Frame-shift
	STX_EG1074P	WS	0.26	
	STX_P_22	NDD	0.1	
	STX_Syrbe_21	WS	3	
c.795-2A>G	STX_25951140_Case_32	EOEE	0.5	In-frame 108bp deletion
	STX_26514728_Patient_4	EOEE	0.35	
c.795-2A>T	STX_26865513_Patient_22	EOEE	2.5	In-frame 12bp deletion
c.1030-1G>A	STX_29896790_P2	EOEE	0	In-frame 81bp deletion

## The overall profile of *STXBP1* non-canonical splice variants

For non-canonical splice variants with a broader range of functional consequences and thus a wider phenotypic spectrum, their contribution to disease is more difficult to establish. Functional characterization is not practical for all of them, so accurate prediction tools (such as SpliceAI) serve as a good approximate proxy. In total, 203 *STXBP1* non-canonical splice variants (synonymous variants close to splice sites and deeper intronic variants) from Clinvar were grouped based on their respective clinical significance. By SpliceAI, 13 out of the 15 pathogenic or likely pathogenic variants (87%), eight out of the 18 variants of uncertain significance (44%), and one out of the four variants with conflicting interpretations of pathogenicity (25%) were predicted with splicing changes that are likely functionally relevant, consistent with the designated clinical significance of each group. From the remaining benign or likely benign variants, we randomly picked 20 for SpliceAI prediction, and only two showed a positive indication for likely significant splicing changes (10%). No detailed clinical data were available from Clinvar for more in-depth phenotypic evaluation. Ultimately, experimental functional characterization is required for confirmation, but prediction tools are quite useful to prioritize a large number of candidate splice variants.

To complement the analysis of data from Clinvar, which is a database for disease-associated variants, 6824 *STXBP1* variants (including deep intronic regions) from gnomAD (15) and the Genome Aggregation Database from population genetic studies were evaluated, and none of the canonical splice variants showed up. In addition, 23 of the 24 non-canonical splice variants that have likely significant splicing changes were absent from the gnomAD database, and only one was reported with one allele out of 2,51,470 (a frequency of 3.98e-06). In contrast, many of the variants with no predicted splicing changes have a high allele frequency in gnomAD.

## *STXBP1* c.578+2T>C and related splice variants

*STXBP1* c.578+2T>C and two splice variants nearby (c.578+1G>A and c.578+1dupG) shared severe splicing defects as predicted by SpliceAI and early onset EOEE in respective patients (Table 2). Similarly, two other +2T>C variants (c.169+2T>C and c.1249+2T>C) were predicted to cause a frameshift, and patients with those variants showed early onset *STXBP1*-related disorders. Previous studies suggested that +2T>C variants typically lead to exon skipping (rather than intron retention) and/or activation of the cryptic splice site (8). However, the RNA analysis of our patient with c.578+2T>C revealed intron retention as the functional consequence. Future investigations on more *STXBP1* +2T>C variants will help clarify a general pattern in terms of their effect on splicing.

Most of the splice variants were reported in only one patient, but there are several associated with multiple patients, such as c.1249+2T>C, c.1359+1G>A, c.1702+1G>A, and c.795-2A>G (Table 2). For those recurrent splice variants, the associated disease categories could be diverse, but the age of disease onset was generally consistent, except for c.1359+1G>A, with which one patient had much later disease onset than the other two patients (72 months compared to 1 and 4 months).

## Discussion

### Genotype–phenotype relationship of *STXBP1*-related disorders

As illustrated in one recent comprehensive profiling of *STXBP1* variants, no significant phenotypic similarity or discrete phenotypic subgroups emerged for recurrent *STXBP1* variants and mutation hotspots due to a high baseline variability (2). Until now, no clear genotype–phenotype correlations for *STXBP1*-related disorders have been identified despite several large-scale efforts (2, 4, 7). However, the majority of patients with *STXBP1*-related disorders present with neurodevelopmental abnormalities (developmental delay and intellectual disability) and seizures (mostly onset in the first year of life) across the whole spectrum of many different types of genetic variants (missense, nonsense, frameshift, and splice variants, small intragenic deletions and duplications, and whole-gene deletions) (1, 2, 4), suggesting an overall genotype–phenotype correlation and shared overarching disease mechanisms that are consistent with the molecular function of STXBP1 protein during synaptic transmission. In addition, monozygotic twins with the same *STXBP1* splice variant presented with similar phenotypes and disease course (7), and two sisters with identical *STXBP1* missense variants showed highly similar clinical symptoms, whereas their heterozygous mother and siblings are asymptomatic (6), indicating the consistent role of genetic factors in *STXBP1-*related disease. Therefore, focusing on the overall profile or key phenotypic indicators, rather than individual phenotypic features, maybe a more productive approach to assessing its genotype–phenotype relationship.

Another recent study showed that despite no clear genotype–phenotype correlation, age at the seizure onset correlated with the severity of the developmental outcome, with an earlier seizure onset related to worse developmental achievement (7). Therefore, we focused on the general categories of disease (EOEE, WS, OS, other DEE, or NDD) and the age of seizure onset as key indicators and reassessed several recurrent missense variants reported previously (2). Moreover, different variants at the same site were analyzed separately rather than being grouped as in previous studies (2, 7), with the reasoning that different substitutions at the same position would present with different degrees of functional perturbation due to the varying biochemical properties of different amino acid residues.

Disease categories and ages of seizure onset were analyzed for the top seven missense variants, and a summary is given in Supplementary Table 4. As expected, diverse phenotypes of each variant spanned different categories of *STXBP1*-related disorders, but the ages of seizure onset were all below 1 year with no more than two exceptions for each variant (2/19 for Arg406His, 2/19 for Arg406Cys, 2/18 for Arg292His, 1/10 for Arg292Cys, 2/18 for Arg551Cys, 1/12 for Pro139Leu, and 1/11 for Arg190Trp).

For typical genotype–phenotype analysis beyond recurrent variants, certain types of variants, such as non-sense, splice-site, frameshift, and whole/partial deletion variants, were grouped together as protein-truncating variants due to their presumed similarity in their functional consequences (2). However, this heterogenous group of variants can lead to drastically different outcomes in terms of functional changes that are sufficient to warrant different phenotypes. One recent study of two protein-truncating *STXBP1* variants showed that one deletion variant caused non-sense-mediated decay (NMD) and likely no production of truncated proteins, whereas NMD was not observed with the other non-sense variant, and truncated proteins were possibly produced (9). This different molecular outcome may explain the different disease phenotypes associated with the two variants (seizure-free compared to early onset epileptic spasms). Therefore, more precise classification of variants should not be based on gross groups but instead on efficient and feasible functional assays, which are not always available currently.

On the contrary, different types of variants can lead to similar clinical phenotypes. For example, homozygous *STXBP1* missense variants (p.Leu446Phe) lead to refractory Lennox-Gastaut syndrome and severe intellectual disability (ID) (6), which could also result from a small intragenic deletion of one amino acid residue in another case (p.Lys21del) (2). In comparison, a different small in-frame deletion near p.Lys21 (c.57_59del:p.19_20del) identified in three unrelated patients showed consistent NDD with only mild ID and similar ages of seizure onset (2), arguing for the importance of exact locations even for the same type of variants that are close to each other. The pathogenicity of these variants, as well as the recurrent missense variants described earlier and other examples not listed here, should all be traced back to the biochemical function of respective sites, but only a rather limited number of them have been functionally characterized, such as p.Leu446Phe and p.39dup (6, 16).

With respect to *STXBP1* splice variants, the vast majority of canonical ones correlated well with severe phenotypes and early seizure onset in the patients (Table 2 and Supplementary Table 3). In contrast, non-canonical splice variants form a much larger and more diverse group in terms of their functional consequences and associations with disease (Supplementary Table 2). Despite limited experimental assessments of those variants, accurate *in silico* tools such as SpliceAI, could be utilized for preliminary evaluation and prioritization to facilitate genetic diagnosis. Hopefully, increasing the application of genomic sequencing as the first-line diagnosis tool will enable the identification of more disease-related *STXBP1* variants and together with better reliable predictive and experimental tools to characterize the actual functional consequences, a genotype–phenotype correlation may emerge (4).

### Treatment of *STXBP1-*related disorders

Due to the limited understanding of the disease mechanism and diverse spectrum of both genotypes and phenotypes for *STXBP1*-related disorders, current treatments are focused on seizure control, wherein usually multiple antiepileptic drugs were prescribed, but a significant portion of the patients still had frequent seizures (1, 2, 7). Different drugs showed significantly different efficacies, depending on seizure type and age, but in general, ACTH and phenobarbital were effective in initially decreasing seizure frequency in infantile spasms and focal seizures, whereas the ketogenic diet was the most effective treatment to maintain seizure freedom (2). In our case, ACTH was first used with poor outcomes and concurrent severe pulmonary infections, while benzodiazepines (midazolam and clonazepam) showed good efficacy in seizure control and maintenance, suggesting that individual differences should be taken into consideration for disease management.

Symptom-based seizure control was able to improve behavioral and interactive skills, while additional physical and occupational therapies could relieve locomotor problems and maximize developmental potential (1, 7). However, the currently available multidisciplinary treatments had a minimal impact on global development (7). As illustrated in our patient, the mental and motor development was markedly behind children of the same age and significant intellectual disability persisted despite good seizure control with medication (Table 1 and Supplementary Table 1). A future mechanism-based targeted approach may benefit both seizure and developmental outcomes (1). For example, several chemical chaperons demonstrated a good rescue effect on functional deficits caused by *STXBP1* variants with decreased stability and increased aggregation in multiple *in vitro* and *in vivo* models (5).

## Conclusion

Ohtahara syndrome is an early-onset epileptic encephalopathy with severe psychomotor development delay, characterized by frequent and uncontrollable tonic-spasmodic seizures and periodic burst-inhibition patterns in the EEG of both waking and sleeping phases. For infants with neonatal-onset OS who have neither history of hypoxic-ischemic encephalopathy nor obvious abnormal brain MRI, genetic factors such as *STXBP1* variants should be considered. The identified novel *STXBP1* splice variant in our patient (NM_001032221.4:c.578+2T>C) and the detailed documentation of clinical phenotypes and disease management would enrich the spectrum of genotypes and phenotypes of *STXBP1*-related disorders. A review of *STXBP1* splice variants was also provided to facilitate the understanding of the disease genotype–phenotype relationship.

## Data availability statement

The datasets presented in this article are not readily available because of ethical and privacy restrictions. Requests to access the datasets should be directed to the corresponding authors.

## Ethics statement

This study was approved and carried out under the ethical guidelines for clinical investigation of our institution. Informed consent was obtained and available upon request. Written informed consent was obtained from the participant/patient(s) for the publication of this case report.

## Author contributions

HW and MH: preparing the original draft. HW, MH, and ZZ: reviewing, editing, supervision, and conceptualization. HW, XL, and MH: genetic data analysis. HW, XC, ZL, CC, and ZZ: clinical data acquisition. All authors contributed to the article and approved the submitted version.
