# Supplementary material for: Case report: A novel STXBP1 splice variant and the landscape of splicing-involved STXBP1-related disorders
Source: Front Neurol. 2023 Mar 28;14:1146875. doi: 10.3389/fneur.2023.1146875 (PMC10086123; doi:10.3389/fneur.2023.1146875)
Supplement: Supplementary file 2 [file Data_Sheet_1.docx]

**Supp Table 1. Developmental assessments of the patient**.

| **Age** | **2 months/9 weeks** | **4 months/18 weeks** | **6 months/26 weeks** |
| --- | --- | --- | --- |
| Head circumference (cm) | 39 | 40.5 | 41 |
| Height (cm) | 58 | 61 | 65 |
| Weight (kg) | 5.3 | 5.5 | 6.3 |
| Suck | Yes | Not well | No |
| Chase light/subject | No | No | No |
| Raise the head | / | No | No |
| Turn over | / | No | No |
| Sit | / | / | No |
| **Developmental Quotient (DQ)** | **0.5278** | **0.3248** | **0.2448** |
| Gross motor | 5W/0.5737 | 6W/0.3360 | 7W/0. 2677 |
| Fine motor | 4W/0.4590 | 6W/0. 3360 | 7W/0.2677 |
| Cognitive | 4W/0.4590 | 5W/0.2800 | 6W/0.2295 |
| Language | 6W/0.6885 | 6W/0. 3360 | 6W/0. 2295 |
| Social/emotional | 4W/0. 4590 | 6W/0. 3360 | 6W/0. 2295 |
| Initial diagnosis | Moderate development delay | Severe development delay | Extremely severe development delay |

**Supp table 2. Analysis of *STXBP1* splice variants from Clinvar** (**in attached excel).**

**Supp table 3. Analysis of *STXBP1* canonical splice variants**

| **Variant** | **Patient ID** | **Disease categories** | **Disease onset/Month** |
| --- | --- | --- | --- |
| c.87+1G>T | STX_HSJD_Patient_5 | EOEE | 0.1 |
| c.169+1G>A | STX_19557857_Patient_1 | NDD | 1.38 |
| c.325+1G>T | STX_HSJD_Patient_14 | EOEE | 0.33 |
| c.429+1G>C | STX_G3_P34 | other DEE | 2 |
| c.429+1G>A | STX_20876469_Patient_6 | EOEE | 1 |
| c.663+1G>C | STX_EG0542P | EOEE | 0.13 |
| c.663+1G>A | STX_26384463_Patient_2 | OS | 0.07 |
| c.794+1G>T | STX_BCH_004 | EOEE | 1 |
| c.902+1G>A | STX_21770924_Patient_1 | EOEE | 0 |
| c.1029+1G>T | STX_20876469_Patient_3 | EOEE | 0 |
| c.1461+1G>A | STX_Syrbe_17 | Other DEE | 14 |
| c.1462-2A>T | STX_23533165_Patient_1 | EOEE | 1.38 |
| c.1547+1G>A | STX_G3_P38 | other DEE | 2 |

**Supp Table 4. Reassessment of recurrent *STXBP1* missense variants.**

| **Variant** | **Number of patients** | **Disease categories** | **Number of patients with disease onset > 12 months** |
| --- | --- | --- | --- |
| **p.Arg406His** | 19 | EOEE, OS, WS, Other DEE, NDD, atypical Rett syndrome | 2 |
| **p.Arg406Cys** | 19 | EOEE, OS, WS, Other DEE, NDD, atypical Rett syndrome | 2 |
| **p.Arg292His** | 18 | EOEE, WS, NDD, atypical Rett syndrome | 2 |
| **p.Arg292Cys** | 10 | EOEE, WS, Other DEE, NDD | 1 |
| **p.Arg551Cys** | 18 | EOEE, WS, Other DEE, NDD | 2 |
| **p.Pro139Leu** | 12 | EOEE, OS, WS, Other DEE, NDD, atypical Rett syndrome | 1 |
| **p.Arg190Trp** | 11 | EOEE, WS, Other DEE, NDD | 1 |
